# Physical Activity Assessment Using an Activity Tracker in Patients with Rheumatoid Arthritis and Axial Spondyloarthritis: Prospective Observational Study

**DOI:** 10.2196/mhealth.7948

**Published:** 2018-01-02

**Authors:** Charlotte Jacquemin, Hervé Servy, Anna Molto, Jérémie Sellam, Violaine Foltz, Frédérique Gandjbakhch, Christophe Hudry, Stéphane Mitrovic, Bruno Fautrel, Laure Gossec

**Affiliations:** ^1^ Rheumatology Department Pitié Salpêtrière Hospital Assistance Publique-Hôpitaux de Paris Paris France; ^2^ GRC-UPMC 08 (EEMOIS), UPMC Univ Paris 06, Sorbonne Université Paris France; ^3^ Sanoïa, E-Health Services Gardanne France; ^4^ Rheumatology B Department Cochin Hospital Assistance Publique-Hôpitaux de Paris Paris France; ^5^ INSERM (U1153), Clinical Epidemiology and Biostatistics Paris-Descartes University Sorbonne Paris-Cité Paris France; ^6^ Rheumatology Department St-Antoine Hospital Assistance Publique-Hôpitaux de Paris Paris France; ^7^ DHU i2B, INSERM UMRS_938, UPMC Univ Paris 06, Sorbonne Université Paris France

**Keywords:** fitness tracker, exercise, rheumatoid arthritis, axial spondylarthritis

## Abstract

**Background:**

Physical activity can be tracked using mobile devices and is recommended in rheumatoid arthritis (RA) and axial spondyloarthritis (axSpA) management. The World Health Organization (WHO) recommends at least 150 min per week of moderate to vigorous physical activity (MVPA).

**Objective:**

The objectives of this study were to assess and compare physical activity and its patterns in patients with RA and axSpA using an activity tracker and to assess the feasibility of mobile devices in this population.

**Methods:**

This multicentric prospective observational study (ActConnect) included patients who had definite RA or axSpA, and a smartphone. Physical activity was assessed over 3 months using a mobile activity tracker, recording the number of steps per minute. The number of patients reaching the WHO recommendations was calculated. RA and axSpA were compared, using linear mixed models, for number of steps, proportion of morning steps, duration of total activity, and MVPA. Physical activity trajectories were identified using the K-means method, and factors related to the low activity trajectory were explored by logistic regression. Acceptability was assessed by the mean number of days the tracker was worn over the 3 months (ie, adherence), the percentage of wearing time, and by an acceptability questionnaire.

**Results:**

A total of 157 patients (83 RA and 74 axSpA) were analyzed; 36.3% (57/157) patients were males, and their mean age was 46 (standard deviation [SD] 12) years and mean disease duration was 11 (SD 9) years. RA and axSpA patients had similar physical activity levels of 16 (SD 11) and 15 (SD 12) min per day of MVPA (*P*=.80), respectively. Only 27.4% (43/157) patients reached the recommendations with a mean MVPA of 106 (SD 77) min per week. The following three trajectories were identified with constant activity: low (54.1% [85/157] of patients), moderate (42.7% [67/157] of patients), and high (3.2% [5/157] of patients) levels of MVPA. A higher body mass index was significantly related to less physical activity (odds ratio 1.12, 95% CI 1.11-1.14). The activity trackers were worn during a mean of 79 (SD 17) days over the 90 days follow-up. Overall, patients considered the use of the tracker very acceptable, with a mean score of 8 out 10.

**Conclusions:**

Patients with RA and axSpA performed insufficient physical activity with similar levels in both groups, despite the differences between the 2 diseases. Activity trackers allow longitudinal assessment of physical activity in these patients. The good adherence to this study and the good acceptability of wearing activity trackers confirmed the feasibility of the use of a mobile activity tracker in patients with rheumatic diseases.

## Introduction

### Physical Activity Recommendations

Recommendations for management of inflammatory joint diseases, in particular rheumatoid arthritis (RA) and axial spondyloarthritis (axSpA), state that physical activity should be encouraged [1,2]. Physical activity is not only important for general health [3,4] but it also helps to reduce pain and improve quality of life in rheumatic diseases [5,6]. To meet the World Health Organization (WHO) guidelines, a minimum of 150 min per week of moderate activity or 75 min per week of vigorous activity is recommended [7]. These guidelines have been converted in terms of steps per day and correspond, on average, to 7000 to 11,000 steps per day [8]. The threshold of 10,000 steps per day is usually retained for healthy persons [8-10]. The intensity of physical activity may be estimated by the step cadence (number of steps per minute) as follows: 100 and 130 steps per min correspond approximately to moderate and vigorous physical activity, respectively, and a cadence below 20 steps per min is considered as rest [8,9].

### Assessment of Physical Activity in RA and axSpA

From previous published studies, patients with RA and axSpA appear to have low physical activity levels compared with guidelines and to healthy controls [11-16]. For example, a recent study found a median of 3710 steps per day in patients with RA based on a pedometer assessment [15]. However, these studies were cross-sectional and did not use mobile devices. Mobile activity trackers, such as smartwatches or “connected bracelets” (Withings, FitBit, Jawbone, and MisFit), allow both an interactive feedback on physical activity and the visualization of the evolution of precised activity patterns over time [17]. There is a growing interest in their use and their place in the management of chronic conditions [18,19], including in the field of rheumatology for noninflammatory diseases [20]. In patients with inflammatory joint disease, little is known regarding patterns of physical activity. Mobile activity trackers may contribute to determine profiles of patients with inflammatory joint disease for different physical activity patterns [16], according to duration, intensity, and frequency of physical activity.

### Objectives

The primary objectives of this study were to measure and compare physical activity using a mobile activity tracker over 3 months, between RA and axSpA according to different physical activity patterns, including the number of steps and the duration of moderate to vigorous activity; to assess the proportion of patients reaching the recommendations; to determine trajectories of patients with different evolutions of physical activity; and to explore factors associated with a low physical activity. A secondary objective was to assess the feasibility of such a study using a mobile device.

## Methods

### Study Design

The ActConnect study was a prospective, multicenter, pragmatic, longitudinal observational study. It took place in 6 participating centers (3 tertiary care hospitals and 3 private practice physicians’ offices) in Paris, France. All patients received full information at inclusion and provided informed consent. Ethical approval was obtained from the institutional review board (CPP Ile de France VI) and the human research ethics committee (CCTIRS, number 16.057bis).

### Patients and Controls

Patients were eligible if they were above 18 years of age; had definite RA or axSpA according to the American College of Rheumatology/European League Against Rheumatism (ACR/EULAR) classification criteria [21] or to the Assessment of SpondyloArthritis international Society (ASAS) [22] classification criteria for RA and axSpA, respectively; had an Internet access; and if they owned a mobile phone or tablet, which was compatible with the mobile activity tracker. All consecutive patients who satisfied the inclusion criteria, seen as outpatients (either consultation or day care hospital) by one of the investigators in the participating centers, were asked to participate between January 4 and April 29, 2016.

For indicative purposes, 20 healthy controls, with no rheumatic disease and aiming for similar mean age and gender distribution as for patients, were recruited from a convenience sample.

### Data Collection

#### Medical Data

Characteristics of patients with RA and axSpA were collected at baseline and included sex, age, type of arthritis, disease duration (from diagnosis), and ongoing pharmacological arthritis treatment (including biologics and stability of the treatment during the last 3 months).

Comorbidities were collected using the Functional Comorbidity Index, which ranges from 0 (0=no comorbidity; however, the minimal score was 1 in this study because of the rheumatic disease) to 18 [23]. Where available, in patients with RA, the status for rheumatoid factor (RF) and for anticyclic citrullinated peptide (anti-CCP) and the presence of radiographic erosions were recorded; in patients with axSpA, the Human Leukocyte Antigen (HLA) B27 status, history of peripheral and of extra-articular symptoms, and the presence of sacroiliitis on x-ray or on magnetic resonance imaging (MRI) were recorded. All imaging data were collected as recorded in the medical files, based on local readings in the context of usual care. Disease activity was assessed by the last available disease activity score 28 (DAS28) in patients with RA and by the Bath Ankylosing Spondylitis Disease Activity Index (BASDAI) on a 0 to 10 scale in patients with axSpA [24-26]; all patients filled in a patient global assessment (PtGA) [27]. Disability was self-assessed at the end of the 3 months using the modified Health Assessment Questionnaire (mHAQ), which ranges between 0 and 3 [28], and using the Bath Ankylosing Spondylitis Functional Index (BASFI) in patients with axSpA.

#### General Data

Patients and controls self-reported weight, height, socioprofessional category, work status, and current use of an activity tracker.

#### Physical Activity Data Collection

Each participant received an activity tracker (Withings Activité Pop [29]) and was instructed to wear it every day for 3 months. The Withings tracker is a watch with analog time display, and it records the number of steps per minute. This device was selected for practical aspects (months of autonomy and waterproof) and precision (steps per minute). The watch needed to be connected by Bluetooth, at least every 2 days, to a mobile phone app, which automatically transferred the physical activity data to the database. Data for 90 consecutive days from the first Monday following activation of the device were collected. No intervention was specifically performed to increase physical activity, and no instruction about physical activity was given to the participants.

#### Definitions and Description of Physical Activity Patterns

Physical activity was assessed by the number of steps per day (from 00:00 h to 23:59 h). Different physical activity patterns were also considered: proportion of morning steps over a day (number of steps between 00:00 h and 12:00 h, over the total number of daily steps) to reflect morning stiffness and activity duration in moderate to vigorous intensity (sum over a day of minutes with at least 100 steps recorded). The total activity duration (sum of minutes with at least 20 steps per min) and the number of steps per day in moderate to vigorous intensity (at least 100 steps per min) were also assessed but were strongly correlated to number of steps per day and to activity duration in moderate to vigorous intensity, respectively. The proportion of patients reaching the WHO recommendations [7] in terms of duration of moderate to vigorous activity per week and according to the threshold of 10,000 steps per day was calculated.

### Acceptability Questionnaire

At the end of the 3 months, all patients answered an acceptability questionnaire prepared for this study. Ten questions were selected based on main barriers that emerged after interviewing 5 patients with RA and 5 patients with axSpA. Questions included, among others, are as follows: difficulties or discomfort to handle the bracelet because of the arthritis, worries about security of the bracelet data, and perceived utility of the activity tracker in daily life (Multimedia Appendix 1).

### Statistical Analysis

#### Sample Size

To detect a difference of 1500 steps per day between patients with RA and axSpA, with a standard deviation (SD) of 3300 [15], and considering a statistical power of 80% and a significance level of 5%, 76 participants had to be analyzable in each group. For indicative purposes, 20 healthy controls were also included as anchor comparators.

#### Study Population

Patients and controls were analyzed only if they wore the bracelet for at least 60 complete days (of the 90). Only “full” days were analyzed (ie, at least 8 h recorded by the tracker between the first and the last steps). For sensitivity analyses, each missing day was imputed for the different activity patterns by the mean of the same weekdays for which data were available.

#### Physical Activity Over 3 Months

Physical activity patterns and their distributions over time were described in patients and controls and were compared between RA and axSpA using linear mixed-effect models. The nlme package in R was used [30]. Models included random intercept and slopes for patients and fixed effect for type of arthritis. The timepoints were each day from day 1 to day 90. When observed, the heteroscedasticity was modeled using the power variance function. Covariates included the following baseline characteristics, which visually differed between groups and/or may impact physical activity: sex, age, body mass index (BMI), disease duration, biologics, employment status, and PtGA. The probability for RA and axSpA patients to reach the WHO recommendations according to their rheumatic diagnosis was assessed by logistic regression adjusted on the same covariates.

#### Trajectories of Physical Activity

We assumed that patients may have different evolution of their physical activity over 3 months; some patients may tend to increase or decrease their physical activity because of the motivational aspect of the activity tracker or because of loss of this motivation. For each physical activity pattern, patients’ trajectories were partitioned using the K-means method adapted to longitudinal data in the R package Kml [31,32]: three clusters of patients with homogeneous trajectories of physical activity were identified. The K-means method is an explanatory analysis and needs no assumption about trajectories before running the algorithm. Different quality criteria allow to select the best partition, that is, the best number of clusters, based on the highest between-cluster covariance (well-separated clusters) and on the lowest within-cluster covariance (compact clusters). To explore factors associated with the low physical activity cluster, a multivariable logistic regression was performed. Covariates included type of arthritis, sex, age, BMI, disease duration, biologics, employment status, and baseline PtGA.

#### Acceptability and Adherence to the Activity Tracker

Acceptability was assessed in all included patients by the mean number of days the tracker was worn over the 3 months (ie, adherence), the percentage of wearing time, and by an acceptability questionnaire.

All analyses were performed using R version 3.2.2 [33].

## Results

### Demographic Characteristics

**Figure 1:**
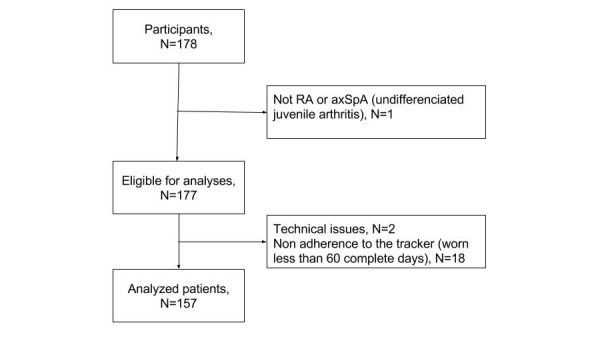
Flowchart of 178 included RA and axSpA patients. RA: rheumatoid arthritis; axSpA: axial spondyloarthritis.

Overall, 178 patients and 20 controls were included. Among them, 157 patients (83 patients with RA and 74 patients with axSpA), with a total of 13,179 days of recording, and 19 controls wore the bracelet for at least 60 complete days and were analyzed (Figure 1). Patients had a mean age of 45.8 (SD 12.5) years, a mean BMI of 25.1 (SD 4.5) kg/m^2^, and a mean disease duration of 10.5 (SD 8.9) years (Table 1). The majority (76.4%, 120/157) of patients were working. Patients with RA were mostly females and older than patients with axSpA; 61% (51/83) had radiographic erosions, and 80% (63/79) had positive RF and/or anti-CCP. In patients with axSpA, 59% (41/70) had past or present peripheral symptoms, 41% (30/73) had experienced extraarticular symptoms, 74% (49/66) were HLA B27 positive, and 82% (55/67) had radiographic and/or MRI sacroiliitis. Disease activity was well controlled (mean PtGA: 3.3 [SD 2.4]; mean DAS28 in RA: 2.3 [SD 1.2]; mean BASDAI in axSpA: 3.2 [SD 2.1]), and many patients were treated with biologics. Patients were little disabled; in RA, mean mHAQ was 0.2 (SD 0.4), and in axSpA, mean mHAQ was 0.3 (SD 0.3) and mean BASFI was 1.7 (SD 1.8). Treatments were globally stable; 19.1% (30/157) patients reported a change over the 3 months in any treatment specific to their arthritis. Controls’ demographic characteristics visually appeared similar to patients (Table 1). The 21 patients not included in the analysis had comparable characteristics to those included (data not shown).

### Physical Activity Over 3 Months

**Figure 2:**
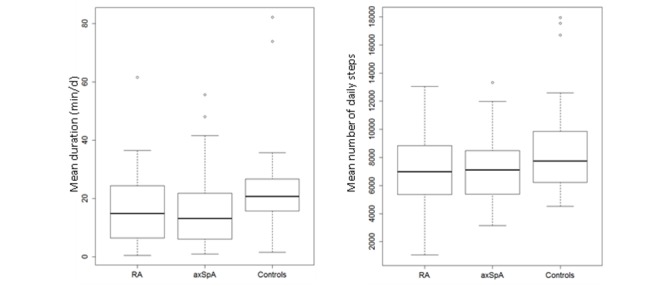
Distribution of physical activity patterns in 83 rheumatoid arthritis and 74 axial spondyloarthritis patients and in 19 controls: (A) mean duration of moderate to vigorous activity (min per day) and (B) mean number of daily steps (steps per day). RA: rheumatoid arthritis; axSpA: axial spondyloarthritis.

**Figure 3:**
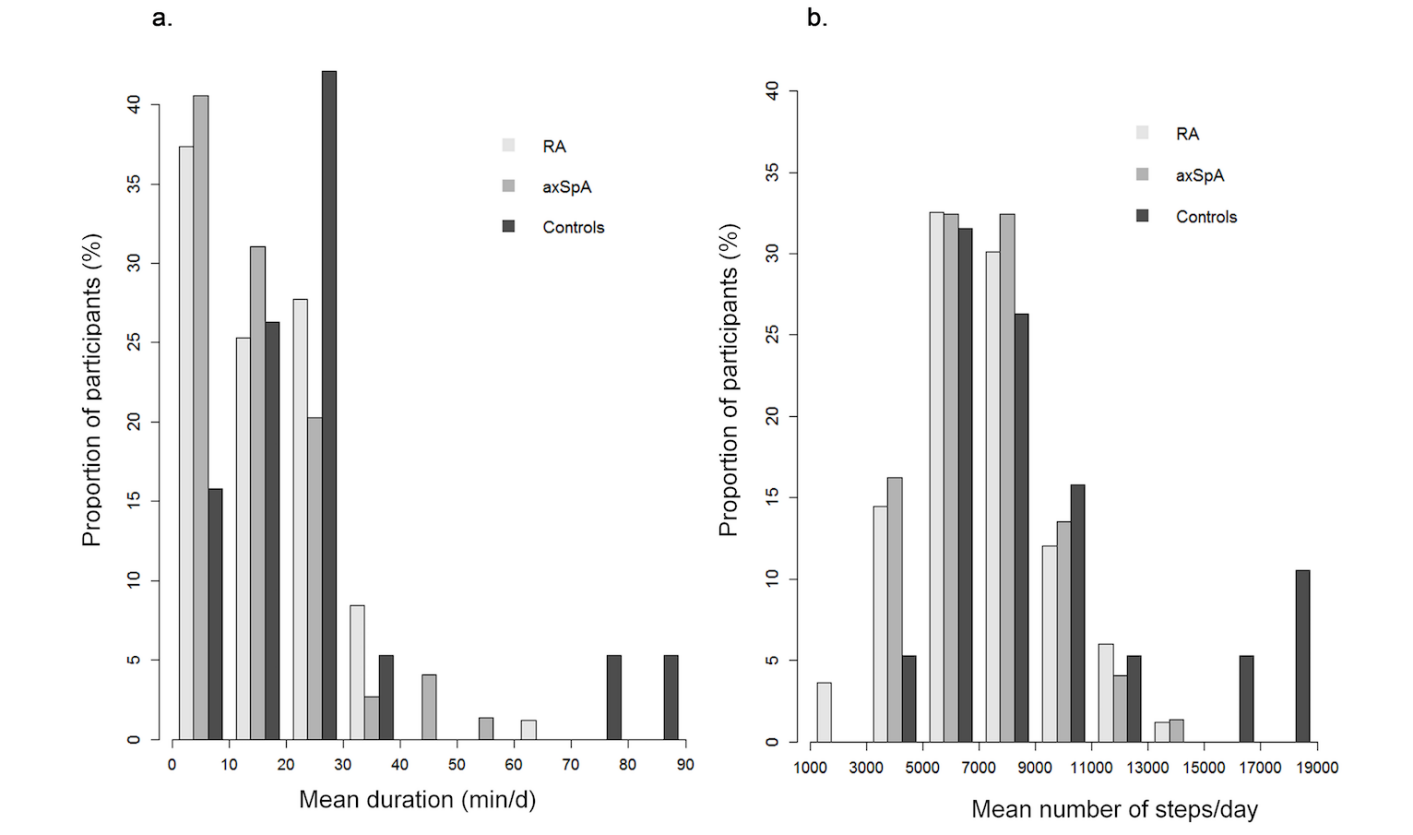
Distribution of mean physical activity over 3 months in 83 RA, 74 axSpA and 19 controls: (A) mean duration of moderate to vigorous activity (min/d) and (B) mean number of steps per day. RA: rheumatoid arthritis; axSpA: axial spondyloarthritis.

Patients performed, on average, 7124 (SD 2316) steps per day, corresponding to 108 (SD 36) min per day of activity, of which 16 (SD 11) min per day (ie, 106 [SD 77] min per week) corresponded to moderate to vigorous intensity (Table 2; Figure 2; Figure 3). Controls tended to be more active than patients with, on average, 9153 (SD 4127) steps per day, corresponding to 132 (SD 60) min per day of activity of which 26 (SD 20) min per day (ie, 174 [SD138] min per week) corresponded to moderate to vigorous intensity (Table 2; Figure 2). Thus, patients had 20% less total activity duration and daily steps compared with controls. This difference increased to 40% for moderate to vigorous activity duration. Overall, 27.4% (43/157) patients reached the WHO recommendations (with a mean of 204 [SD 56] min per week). Fifteen out of 157 (9.6%) patients walked with a mean of more than 10,000 steps per day (Table 2). Eight out of 19 (42%) and 5 out of 19 (26%) controls reached the following recommandations, respectively: mean 150 min per week of moderate to vigorous activity and mean 10,000 steps per day.

In longitudinal analyses, there was no statistically significant difference between patients with RA and axSpA in physical activity levels or patterns (Table 2).

**Figure 4:**
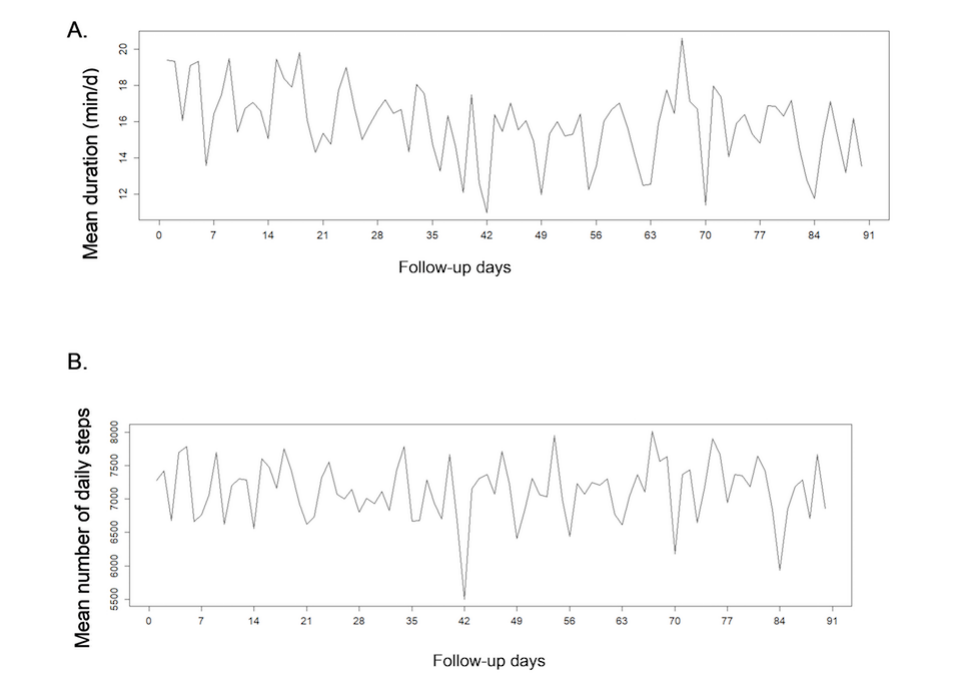
Weekly fluctuations of physical activity in 83 rheumatoid arthritis patients over 90 days, according to: (A) moderate to intense activity duration and (B) number of steps.

**Figure 5:**
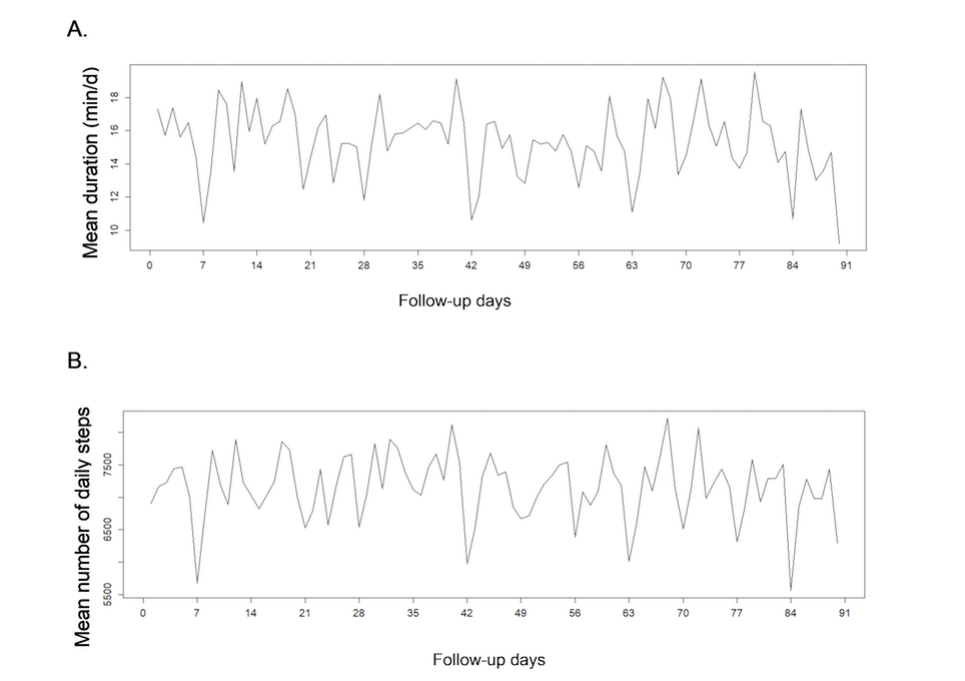
Weekly fluctuations of physical activity in 74 axialspondyloarthritis patients over 90 days, according to: (A) moderate to intense activity duration and (B) number of steps.

The different activity patterns varied across days with periodic weekly variations in patients (Figures 4 and 5) and in controls (data not shown). Sundays seemed to be related to a decrease in all activity patterns (note that days 0, 7, 14, etc represent Sundays in Figures 4 and 5). The mean level of physical activity appeared to remain constant over the 3 months in patients and controls.

**Table 1:** Characteristics of 157 patients and 19 controls participating in an observational study of physical activity using an activity tracker.

Characteristics		RA^a^ (N=83)	axSpA^b^ (N=74)	Controls (N=19)
Sex, males, n (%)		14 (16.9)	43 (58.1)	8 (42.1)
Age in years, mean (SD^c^)		49.9 (12.9)	41.3 (10.4)	45.1 (11.4)
BMI^d^, kg/m^2^, mean (SD)		24.9 (4.4)	25.3 (4.6)	24.2 (4.0)
Disease duration in years, mean (SD)		10.7 (8.8)	10.4 (9.1)	—
**Work status, employed, n (%)**		59 (71.1)	61 (82.4)	18 (94.7)
	Manual work	3 (5.1)	3 (4.9)	0 (0.0)
	Intellectual work	56 (94.9)	58 (95.1)	18 (100.0)
University studies, n (%)		68 (81.9)	62 (83.8)	17 (89)
Physical activity record history, n (%)		14 (16.9)	15 (20.3)	7 (36.8)
Functional comorbidity Index (ranging from 0 to 18), mean (SD)		1.6 (0.9)	1.3 (0.9)	
mHAQ^e^, mean (SD)		0.2 (0.4)	0.3 (0.3)	
PtGA^f^, mean (SD)		3.1 (2.3)	3.7 (2.5)	
Treatment at inclusion NSAIDs^g^, n (%)		18 (21.7)	47 (63.5)	
Oral glucocorticoids, n (%)		21 (25.3)	1 (1.4)	
**Synthetic DMARDs**^h^**, n (%)**		78 (94.0)	17 (23.0)	
	Methotrexate	68 (87.2)	13 (76.5)	
**Biological therapy, n (%)**		42 (50.6)	46 (62.2)	
	antiTNF^i^	27 (64.3)	46 (100.0)	

^a^RA: rheumatoid arthritis.

^b^axSpA: axial spondyloarthritis.

^c^SD: standard deviation.

^d^BMI: body mass index.

^e^mHAQ: modified Health Assessment Questionnaire.

^f^PtGA: patient global assessment.

^g^NSAIDs: nonsteroidal antiinflammatory drugs.

^h^DMARDs: disease modifying antirheumatic drugs.

^i^antiTNF: antitumor necrosis factor.

**Table 2:** Physical activity patterns in RA and axSpA patients.

Physical activity patterns	RA^a^ (N=83)	axSpA^b^ (N=74)	*P* value^c^	Controls (N=19)
Total activity duration (min/day)^d^, mean (SD^e^)	108 (39)	108 (31)	.51^f^	132 (60)
Activity duration/day in moderate to vigorous intensity (min/day)^g^, mean (SD)	16 (11)	15 (12)	.80^f^	26 (20)
Number of steps/day, mean (SD)	7118 (2411)	7130 (2221)	.50^f^	9153 (4127)
Percentage of steps before 12 AM, mean (SD)	32 (8)	32 (7)	.53^f^	36 (6)
Participants fulfilling the WHO^h^ recommendation (mean physical activity≥150 min/week of moderate to vigorous activity), n (%)	25 (30)	18 (24)	.76^i^	8 (42)
Participants fulfilling the mean physical activity of ≥10,000 steps/day recommendations, n (%)	9 (11)	6 (8)	.71^i^	5 (26)

^a^RA: rheumatoid arthritis.

^b^axSpA: axial spondyloarthritis.

^c^*P* value of the comparison between RA and axSpA patients.

^d^At least 20 steps per min.

^e^SD: standard deviation.

^f^Comparison between RA and axSpA using linear mixed models adjusted on baseline characteristics.

^g^At least 100 steps per min.

^h^WHO: World Health Organization.

^i^Comparison between RA and axSpA using logistic regression adjusted on baseline characteristics.

### Comparisons of Clusters of Patients According to Their Trajectories

**Figure 6:**
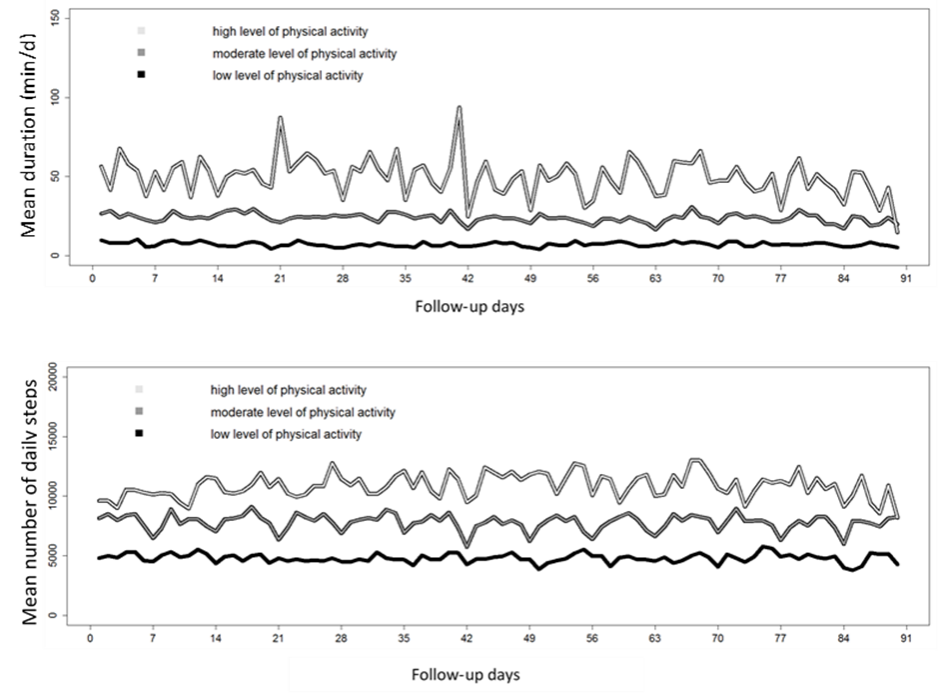
Mean trajectories over 3 months of all patients partitioned in 3 clusters according to: (A) moderate to vigorous activity duration and (B) number of steps per day.

Patients were partitioned in 3 clusters with homogeneous trajectories according to following 3 levels of activity: low, moderate, and high (Figure 6). In all clusters, trajectories were overall constant over time.

Considering the duration of moderate to vigorous activity (Table 3), 54.1% (85/157) patients had a low activity level (mean of 7.2 [SD 4.2] min per day), 42.7% (67/157) had a moderate activity level (mean of 23.8 [SD 5.3] min per day), and 3.2% (5/157) had a high activity level (mean of 49.4 [SD 9.2] min per day). Patients with RA in the low activity cluster (n=44) had a mean DAS 28 of 2.1 (SD 0.9) versus 2.6 (SD 1.4) in the moderate to high cluster (n=39). Patients with axSpA had, respectively, a BASDAI of 2.7 (SD 2.1) and 3.6 (SD 2.0) in the low activity cluster (n=41) and the moderate to high activity cluster (N=33). The main factor associated with being in the low activity cluster was a higher BMI (odds ratio [OR] for a 5-point increase, OR 1.13, CI 1.11-1.15, *P*=.007).

Considering the number of steps per day (Table 3), 38.2% (60/157) patients were in the low activity cluster (mean of 4823 [SD 1020] steps per day), 46.5% (73/157) were in the moderate activity cluster (mean of 7789 [SD 877] steps per day), and 15.3% (24/157) were in the high activity cluster (mean of 10,852 [SD 1259] steps per day). BMI and biological therapy were associated with the low activity cluster.

In sensitivity analyses, all results were confirmed after imputation of missing days (data not shown).

### Acceptability of the Activity Tracker

For the 177 eligible patients (Figure 1), the activity trackers were worn during a mean of 79 (SD 17) days, corresponding to a mean of 88% (SD 19) of days over the period; 70.6% (125/177) patients wore it for at least 80 out of 90 days, and 78.5% (139/177) patients still wore the device at the end of the 3-month period. Patients (N=171) considered the use of the watch very acceptable, with a mean score of 8 out 10, and self-reported barriers were rare (Multimedia Appendix 1). At the end of the study, 63.2% (108/171) patients were considering to keep wearing the tracker most of the time. Only 4.5% (8/177) patients (4 with RA and 4 with axSpA) reported to have problem with the watch because of their arthritis. When the adherent patients (ie, analyzed ones, N=153) were compared with the nonadherent patients (N=18) for acceptability, adherent patients tended to find the tracker slightly more acceptable; the mean acceptability scores were 8.6 out 10 (SD 2.4) and 7.8 out 10 (SD 2.8), respectively; and 65% versus 50% of adherent versus nonadherent patients considered to keep wearing the tracker regularly after the end of the study.

**Table 3:** Comparison between patients in the low activity cluster and other patients.

Factors associated with a low activity	Clusters according to moderate to vigorous activity duration				Clusters according to number of steps per day			
	Low cluster (N=85)	Other clusters (N=72)	OR^a^ (95% CI)	*P* value	Low cluster (N=60)	Other clusters (N=97)	OR (95% CI)	*P* value
Type of arthritis, axSpA^b^, n (%)	41 (48)	33 (46)	0.94 (0.79-1.12)	.51	27 (45)	47 (48)	0.97 (0.82-1.15)	.74
Sex, female, n (%)	49 (58)	51 (71)	0.86 (0.72-1.02)	.09	39 (65)	61 (63)	1.00 (0.84-1.18)	.97
Age in years, ≥60 years, n (%)	16 (19)	6 (8)	1.12 (0.84-1.49)	.44	15 (25)	7 (7)	0.97 (1.00-1.69)	.09
BMI^c^, kg/m^2^, mean (SD^d^)	26.1 (5.0)	23.9 (3.5)	1.13 (1.11-1.15)^e^	*.007*^f^	26.5 (5.4)	24.2 (3.5)	1.12 (1.11-1.14)^e^	*.007*
Disease duration, years, mean (SD)	12.3 (9.2)	8.5 (8.2)	1.01(1.00-1.02)	.08	12.1 (9.0)	9.6 (8.8)	1.00 (0.99-1.01)	.79
Employment status, employed, n (%)	63 (74)	57 (79)	1.04 (0.84-1.29)	.74	40 (67)	80 (82)	0.97 (0.78-1.19)	.74
Biological treatment, present, n (%)	54 (64)	34 (47)	1.10 (0.94-1.30)	.23	41 (68)	47 (48)	1.18 (1.01-1.38)	*.04*
PtGA^g^ at baseline, mean (SD)	3.6 (2.5)	3.0 (2.2)	1.02 (0.99-1.06)	.15	3.7 (2.5)	3.1 (2.3)	1.02 (0.98-1.05)	.32

^a^OR: odds ratio of low versus moderate to high activity levels.

^b^axSpA: axial spondyloarthritis.

^c^BMI: body mass index.

^d^SD: standard deviation.

^e^OR expressed for an increase in BMI of 5 units,

^f^*P* value ≤.05 are in italics.

^g^PtGA: patient global assessment.

## Discussion

### Principal Findings

This study brought interesting and original information by exploring longitudinally and objectively physical activity in RA and axSpA using a mobile device. Levels of physical activity were similar in patients with RA and in patients with axSpA, both regarding total and moderate to vigorous physical activity (MVPA), but only infrequently attained the levels recommended by the WHO [7]. A higher BMI was the main factor related to a low level of physical activity. Overall, measuring physical activity using a mobile activity tracker in patients with inflammatory joint disease appeared feasible and acceptable.

### Strengths and Weaknesses

This study has strengths and weaknesses. The patients’ population may not be representative as indicated by high schooling and job levels and by wide use of biologics treatment. However, this may be an inherent bias when studying mobile devices that necessitate recent and powerful mobile phones [29]. Moreover, the high number of included patients makes this study one of the most ambitious studies using a mobile device in the rheumatology field [34]. The control group was included for indicative purposes only, and therefore, was of small size and did not allow statistical comparisons. However, physical activity in controls was close to the general French population activity [35], which tends to strengthen the validity of the physical activity assessment.

Physical activity may have been misjudged. Although patients were not instructed to perform more physical activity, they get feedback on their activity and may have increased it, because of the motivational aspect of the activity tracker in itself [19,36]. However, the longitudinal analyses did not confirm this bias; we would anticipate that this effect would have decreased over 3 months because of progressive loss of motivation, whereas all trajectories were stable over time. The K-means method used to identify trajectories may nevertheless have underestimated changes [37]. However, no assumptions about trajectories are needed before running the algorithm. Another limitation of the K-means method is to find “spherical” clusters, which have a similar size or variance. In case the population comprised groups with different variances, the K-means method would have difficulty identifying the correct clusters. Analyses may also have been biased by different factors, which may influence physical activity, such as comorbidities, and the type of job. However, patients had very few comorbidities, and only few patients had a manual work. Finally, the alpha risk was not controlled, which may be a limitation of the analysis.

Although activity trackers have been validated in other studies [38,39], estimation of physical activity using a mobile tracker may have some limitations. Nonwalking activities, such as cycling, bodybuilding, and dancing, may have been underestimated. However, this bias is inherent to the measuring method as these activities do not imply a number of steps. Conversely, physical activity may have been overestimated by the tracker (eg, because of arm movement), and some false positive steps may have been recorded [40]. The use of a mobile activity tracker may also be limited by the need of owning a recent mobile phone, having Internet access, and being a minimum familiar with technology.

This study explored different activity patterns. The proportion of morning steps was an original pattern, aiming to reflect the morning stiffness. Unfortunately, this analysis was hampered by the difficulty of defining a precise wake-up time. Morning stiffness may directly influence physical activity by delaying the beginning of daily activities, but in this study, as wake-up time was not recorded, it was difficult to analyze the potential reduction of physical activity just after waking up. The detection of morning stiffness using mobile devices should be further explored.

### Which Physical Activity Level to Recommend?

We evidenced that insufficient levels of physical activity were performed both for total physical activity (including slow walking) and MVPA. This indicates that patients with inflammatory joint disease may be both not walking enough and not performing enough aerobic exercises. These findings were confirmed when comparing patients with RA and axSpA with the small sample of healthy persons included. Patients had 20% less total activity duration and daily steps compared with controls. This difference increased to 40% for moderate to vigorous activity duration. However, intense physical activity may not always be increased because of medical issues or aging. It may be more relevant to promote the increase of the total amount of physical activity, including with a low level. Increasing evidence suggests that the practice of all types of physical activity, including low intensity ones, is beneficial to health [41]. Targeting an increase in low to moderate physical activity, such as walking, appears reasonable in patients with rheumatic diseases [42]. These are important data to take into account when discussing physical activity with our patients.

Although the WHO recommendations rest on minutes per week of activity, the message is often transformed into 10,000 steps per day (particularly, when using activity trackers). We show here that these objectives are not equivalent. In this study, 27% of patients (vs 42% of controls) were active enough according to the WHO recommendations (in terms of duration per week of moderate to vigorous activity), but only 10% of patients were above the threshold of 10,000 steps per day. The assessment of physical activity in minutes per week of moderate to vigorous activity may be an achievable and motivating objective for patients with inflammatory joint disease. Activity tracker companies may want to revisit their presentation of physical activity achievements (eg, on their apps). The threshold of both these recommendations may also be too much elevated and difficult to reach, even for healthy persons. Indeed, studies indicate that 58% and 65% of the French and the European general populations, respectively, perform enough physical activity [35,43]. The threshold of 7000 to 8000 steps per day could be more appropriate in elderly or disabled persons [44]. When considering the threshold of 7000 steps per day, 50.3% of the patients would have fulfilled the recommendations. In previous studies, levels of physical activity in patients with inflammatory joint disease were highly variable [11,15,16,45]. Some studies found that more patients were in accordance with the WHO recommendations [11,14,16]. Methods used to assess the intensity of physical activity varied across studies. This study estimated physical activity from the number of steps per minute, whereas in previous studies, the intensity was mostly estimated from energy expenditure (from questionnaires [46] or multisensor devices [11,45]) or from counts per minute (from conventional accelerometers) [12,16]. Moreover, previous studies were conducted over short durations, and participants may have substantially increased their physical activity during those studies.

Similar levels of physical activity were found in RA and axSpA after adjustment on sex and age. A lower level of physical activity may have been expected in RA patients. Indeed, RA frequently affects the feet, which may directly impact physical activity. These surprising results may be partly explained by the fact that the studied population had a stable disease, was frequently treated with biologics, and had good physical function. Due to its subject, this study may have selected less severe patients. Nevertheless, it is interesting to note that physical activity should be encouraged in both patients with RA and with axSpA.

### Risk Factors for a Low Physical Activity

The only factor explaining a low physical activity level in these analyses was a higher BMI. Previous studies found various relations between physical activity and BMI [46,47] and highlighted other factors of inactivity, such as female sex, older age, and lack of motivation [32,48]. Our results suggest that interventions to promote physical activity should target overweight patients. However, given demographic variables did not explain low physical activity, all patients should be encouraged to increase their physical activity. Factors related to a low physical activity level were not explored separately in RA and axSpA subgroups, particularly for disease-specific scores (eg, BASDAI or DAS28). However, physical activity was similar in both subgroups, and the type of arthritis was not related to the low physical activity cluster. The link between physical activity and disease activity should be further studied.

### Feasibility and Acceptance of the Use of an Activity Tracker

Finally, this study demonstrated the feasibility of wearing a mobile device over 3 months in patients with inflammatory joint disease. Although patients with inflammatory joint disease may encounter difficulties that are specific to their disease (eg, to handle the device may be difficult because of hand arthritis) [49], patients wore the device 88% of the days, and many were considering pursuing its use. This study indicates that there are no specific barriers linked to RA or axSpA for using activity trackers. Patients were considered adherent enough to be analyzed if at least 60 out 90 days of physical activity were recorded. This cutoff was selected based on a balance between representativeness of physical activity in each patient and representativeness of the study population. However, nonadherence to the activity tracker was not negligible, leading to 11% of patients not being analyzed. This may reflect the constraint of wearing a mobile device every day over a long period of time. Contrary to conventional accelerometers, mobile activity trackers may appear more acceptable to wear over time. Their availability, their ease of use, and their interactive and playful interface make them good devices to measure physical activity in patients [50]. Activity trackers should be further explored as motivational tools to enhance physical activity in inflammatory joint diseases, as indicated in other chronic conditions [19].

### Conclusion

Patients with RA and axSpA do not perform enough physical activity. Only 27% of patients met the WHO recommendations, when assessed with an activity tracker. The objective of 150 min per week of MVPA appears more feasible than the objective of 10,000 steps per day. Patients should be encouraged to perform more physical activity in line with the WHO recommendations. Patients with RA and axSpA had similar levels of physical activity both regarding total and moderate/vigorous physical activity, recorded with a mobile activity tracker, despite the differences between these two diseases. This study has shown the feasibility and the interest of mobile devices in rheumatology research; longer-term studies are needed.

## Appendix

Multimedia Appendix 1 Acceptability regarding use of a connected activity tracker: questionnaire derived from 10 interviews of RA and axSpA patients and results.

## Abbreviations

ACR/EULAR: American College of Rheumatology / European League Against Rheumatism
anti-CCP: anticyclic citrullinated peptide
ASAS: Assessment of SpondyloArthritis international Society
axSpA: axial spondyloarthritis
BASDAI: Bath Ankylosing Spondylitis Disease Activity Index
BMI: body mass index
DAS: disease activity score
mHAQ: modified Health Assessment Questionnaire
MRI: magnetic resonance imaging
OR: odds ratio
PtGA: patient global assessment
SD: standard deviation
RA: rheumatoid arthritis
RF: rheumatoid factor
WHO: World Health Organization
