# Supplementary material for: Physical Activity Assessment Using an Activity Tracker in Patients with Rheumatoid Arthritis and Axial Spondyloarthritis: Prospective Observational Study
Source: JMIR Mhealth Uhealth. 2018 Jan 2;6(1):e1. doi: 10.2196/mhealth.7948 (PMC5770578; doi:10.2196/mhealth.7948)
Supplement: Multimedia Appendix 1 [file mhealth_v6i1e1_app1.pdf]

Acceptability regarding use of a connected activity tracker: questionnaire derived from 10 interviews of RA and axSpA patients and results

Questions	Patient's answers (n= 171)
1. You have been wearing a connected watch for 3 months for the study: Do you think the use of this watch is acceptable for you? (consider the use of the watch in all its aspects and the hardship that you might have encountered)<br>0 ———————— 10<br>Not acceptable at all — Totally acceptable	Score of acceptability, Mean (SD):<br>8.5 (2.5)
2. Do you plan to keep wearing the watch after the end of the study?<br>❑ Yes, all the time or most of the time<br>❑ Yes, occasionally<br>❑ No	Most of the time, N (%):<br>108 (63)
3. Did you encounter any technical issues to set up the watch and/or the application and to synchronize the watch?<br>❑ No, I did it by myself without any issue<br>❑ Yes, it was difficult but I did it by myself<br>❑ Yes, I needed help	Needed help, N (%):<br>31 (18)
4. Did your arthritis pose problem:<br>❑ To wear the watch<br>❑ To handle the watch or the application<br>❑ No, not for this study	Problem to wear the watch, N (%):<br>8 (5)
5. Generally, is physical activity an important matter for you?<br>❑ No<br>❑ Yes	Yes, N (%):<br>160 (94)
6. Do you think the use of the watch allowed you to increase your physical activity?<br>❑ No<br>❑ Yes	Yes, N (%):<br>107 (63)
7. Do you think measuring the number of steps generally reflects your daily physical activity?<br>❑ No<br>❑ Yes	Yes, N (%):<br>122 (71)
8. Do you feel the watch-recorded activity didn't match some of your activities?<br>❑ No, it generally matched<br>❑ Yes, the watch tends to over-estimate my activity<br>❑ Yes, the watch tends to under-estimate my activity	Physical activity underestimated, N (%):<br>52 (30)
9. In general, do you worry your personal data, when recorded by a connected device, may become public (eg by hacking)?<br>❑ No<br>❑ Yes	Yes, N (%):<br>24 (14)
10. Would you be interested in using a connected device as part of your medical care?<br>❑ No<br>❑ Yes	Yes, N (%):<br>152 (89)

RA: rheumatoid arthritis, axSpA: axial spondyloarthritis, SD: standard deviation
